# Intravenous tPA Therapy Does Not Worsen Acute Intracerebral Hemorrhage in Mice

**DOI:** 10.1371/journal.pone.0054203

**Published:** 2013-02-08

**Authors:** Christian Foerch, Nathanael L. Rosidi, Frieder Schlunk, Arne Lauer, Flor A. Cianchetti, Emiri Mandeville, Ken Arai, Kazim Yigitkanli, Xiang Fan, Xiaoying Wang, Klaus van Leyen, Helmuth Steinmetz, Chris B. Schaffer, Eng H. Lo

**Affiliations:** 1 Neuroprotection Research Laboratory, Massachusetts General Hospital, Harvard Medical School, Boston, Massachusetts, United States of America; 2 Department of Neurology, Johann Wolfgang Goethe-Universität, Frankfurt am Main, Germany; 3 Department of Biomedical Engineering, Cornell University, Ithaca, New York, United States of America; University of South Florida, United States of America

## Abstract

Tissue plasminogen activator (tPA) is the only FDA-approved treatment for reperfusing ischemic strokes. But widespread use of tPA is still limited by fears of inadvertently administering tPA in patients with intracerebral hemorrhage (ICH). Surprisingly, however, the assumption that tPA will worsen ICH has never been biologically tested. Here, we assessed the effects of tPA in two models of ICH. In a mouse model of collagenase-induced ICH, hemorrhage volumes and neurological deficits after 24 hrs were similar in saline controls and tPA-treated mice, whereas heparin-treated mice had 3-fold larger hematomas. In a model of laser-induced vessel rupture, tPA also did not worsen hemorrhage volumes, while heparin did. tPA is known to worsen neurovascular injury by amplifying matrix metalloproteinases during cerebral ischemia. In contrast, tPA did not upregulate matrix metalloproteinases in our mouse ICH models. In summary, our experimental data do not support the assumption that intravenous tPA has a deleterious effect in acute ICH. However, due to potential species differences and the inability of models to fully capture the dynamics of human ICH, caution is warranted when considering the implications of these findings for human therapy.

## Introduction

Stroke is a major cause of death and long-term disability worldwide. For ischemic strokes, clots in the brain can be dissolved with recombinant tissue plasminogen activator (tPA) [1]. Timing of tPA application is critically important. The sooner patients receive tPA and reperfuse, the better the odds ratio for improved outcomes. However, in current clinical practice, performing a brain scan is considered indispensable prior to tPA therapy in order to rule out patients with intracerebral hemorrhage (ICH) [2]. This induces a substantial time-to-treatment delay, as tPA cannot be given at the patients’ home or in the ambulance as is the case for myocardial infarction [3].

Of course, tPA is not completely benign. Many experimental studies now show that excessive tPA can amplify excitotoxic neuronal death and promote blood brain barrier injury [4], [5]. But it is important to remember that, even after factoring in rates of complications and side effects, tPA is still clinically effective when given to the right patients at the right time. Yet, tPA usage is still limited to less than 5% of all ischemic strokes today, more than 10 years after FDA approval. From a clinical and practical perspective, the fear of inadvertently administering tPA in ICH is a major factor that limits the use of this important therapy. The widespread assumption that tPA therapy would worsen ICH seems intuitive, but lacks scientific validation. Here, we tested the effects of intravenous tPA therapy in different experimental models of ICH in mice.

## Results

**Figure 1:**
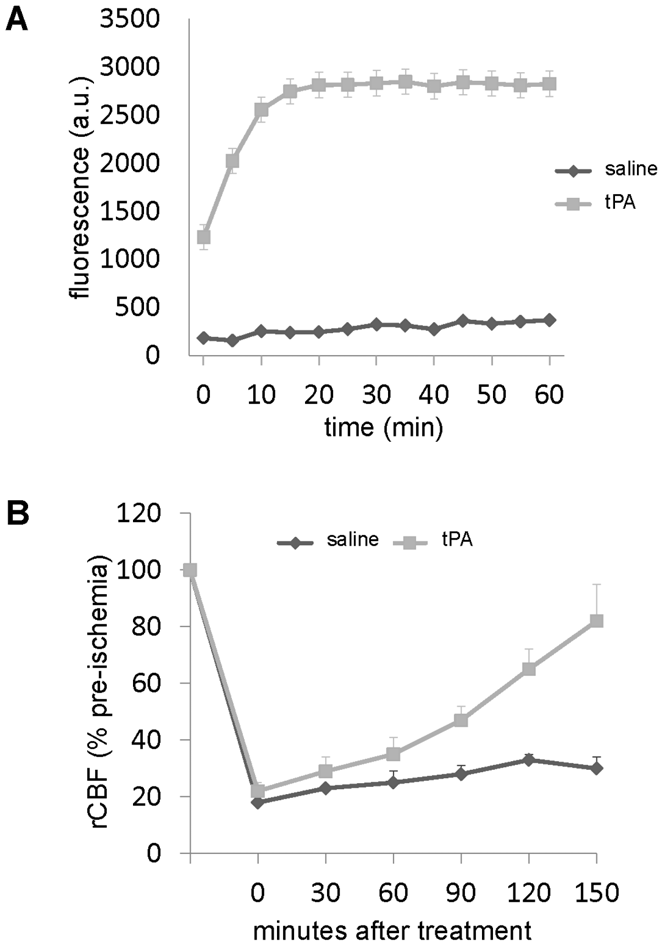
tPA activity measures. (**A**) Human tPA but not saline activates mouse plasminogen. In this assay, tPA converts plasminogen to plasmin, then plasmin converts D-Val-Leu-Lys-7-Amino-4-Methylcoumarin to a fluorescent product, which was measured over 1 hr at 5 minute intervals using a fluorescence plate reader. Data are provided in arbitrary fluorescence units (A.U.). Data are mean±SEM, 3 measures per group and time point. (**B**) Thrombolytic activity of tPA was proven in-vivo in a standard model of thromboembolic middle cerebral artery occlusion in rats. tPA restored regional cerebral blood flow (rCBF) more rapidly than saline. Data are mean±SEM, 4 animals per group.

An in vitro activity assay confirmed that the recombinant human tPA dosing used in our experiments was able to convert mouse plasminogen into active plasmin, the enzyme responsible for clot lysis **(** **Fig. 1A** **)** [6]. Enzyme activity was further confirmed in vivo using a standard rat model of thromboembolic focal cerebral ischemia [7]. Homologous blood clots were intraluminally placed into the middle cerebral artery, and then rats were treated with either saline or 10 mg/kg of tPA at 1 hr post occlusion. Laser Doppler flowmetry confirmed that tPA effectively restored cerebral blood flow **(** **Fig 1B** **)**.

**Figure 2:**
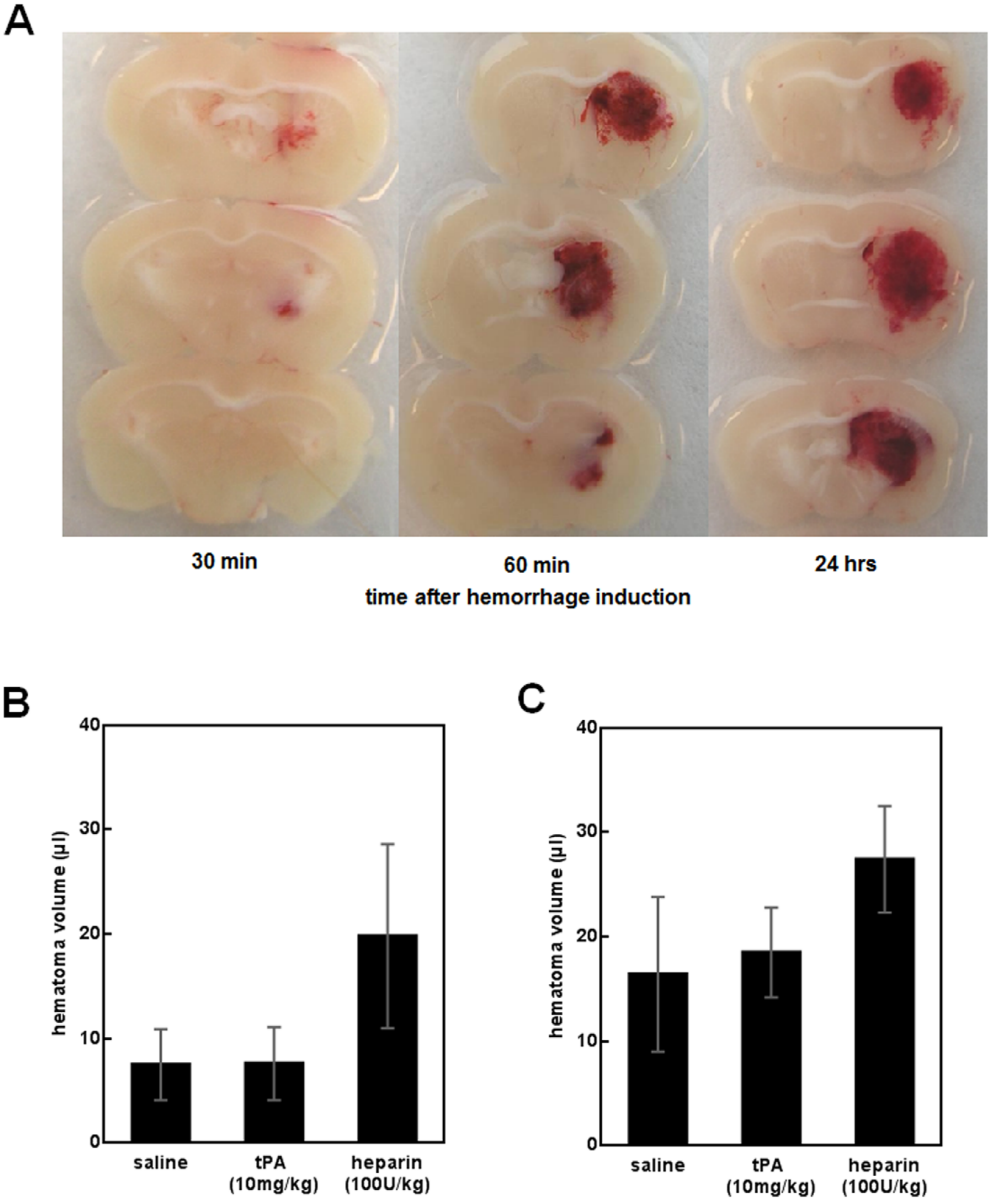
Collagenase-induced ICH volumes are not increased by administration of i.v. tPA. (**A**) Brain sections showing hematoma at 30 min, 60 min and 24 hrs after ICH induction. ICH starts to occur within 30 min and has largely developed by 1 hr. (**B**) Hematoma volumes at 24 hrs after ICH induction (mean±SD). tPA did not alter hematoma sizes, but heparin significantly worsened ICH as compared to saline and tPA treatment. (**C**) Results of the reconfirmation study. No difference was observed between saline and tPA mice, but heparin significantly increased ICH volume (mean±SD) as compared to saline and tPA treatment.

The first model of ICH involved the standard and widely-used stereotactic injection of collagenase type VII-S (0.2 U) into mouse striatum to provoke ICH. Consistent with previous work [8], [9], ICH began within 30 min after collagenase injection, and hematoma development was well underway by 1 hr **(see Methods and** **Fig. 2A** **)**. At 30 min after ICH induction, mice were blindly and randomly assigned to one of 3 treatment groups: saline controls (500 µl, n = 15), tPA (10 mg/kg in 500 µl saline, n = 15), or the anticoagulant heparin (used as a positive control, 100 U/kg in 500 µl saline, n = 4). Treatments were infused over 30 min via a jugular vein catheter. Twenty-four hrs after ICH induction, hematoma volumes were assessed using a photometric assay. Surprisingly, hematoma volumes were not different between saline controls (mean±SD 7.5±3.4 µl) and tPA-treated mice (7.6±3.5 µl), but heparin significantly worsened hemorrhage (19.8±8.8 µl, one-way ANOVA between group differences p<0.001, post-hoc saline vs. tPA p = 1.000, saline vs. heparin p<0.001, tPA vs. heparin p<0.001, **Fig. 2B**). Mortality rate was 0/15 in saline mice, 2/15 in tPA mice, and 2/4 in heparin mice. The two tPA-treated mice that died had pronounced bleeding at the surgical areas (head, neck), but ICH volume was not increased (2.6 and 7.3 µl, respectively). Most likely, death resulted from extracerebral bleeding complications. In contrast, the dead heparin mice had extensive ICH volumes (33.0 and 14.7 µl). The functional impact of ICH, assessed by means of a standard hanging wire test, was not different between saline- and tPA-treated mice **(Fig. S1)**. Because this result was somewhat surprising, a second independent study was initiated to confirm these findings. Using different batches of tPA and collagenase, 24 ICH mice were randomized to saline controls (500 µl, n = 8), tPA (10 mg/kg in 500 µl saline, n = 8), or heparin (100 U/kg in 500 µl saline, n = 8). Once again, treatments were infused over 30 min via a jugular vein catheter. Three mice had to be excluded due to catheter complications (rupture of jugular vein). At 24 hrs, hematoma volumes were generally larger than in the first experimental series, likely due to variations of enzyme activity between different collagenase batches. However, the same pattern of results was obtained between groups. Controls (mean±SD 16.4±7.4 µl) and tPA-treated mice (18.5±4.3 µl) had similar hematoma volumes, whereas heparin-treated mice had larger bleeds (27.4±5.1 µl, one-way ANOVA between group differences p = 0.004, post-hoc saline vs. tPA p = 1.000, saline vs. heparin p = 0.005, tPA vs. heparin p = 0.033, **Fig. 2C**). Mortality rate was 2/7 in saline mice, 4/6 in tPA mice, and 7/8 in heparin mice.

To further confirm these findings, we also tested tPA injection at a later time point (i.e., between 4 and 4.5 hrs after ICH induction when more clot formation in the extravasated blood has occurred). Following the same experimental protocol as above, no difference was found between mice treated with tPA (mean±SD 8.2±5.8 µl) and saline (7.9±2.7 µl, n = 8 per group, t-test p = 0.882), suggesting that no re-bleeding was induced by means of the tPA injection at this later time point after the initiation of ICH.

**Figure 3:**
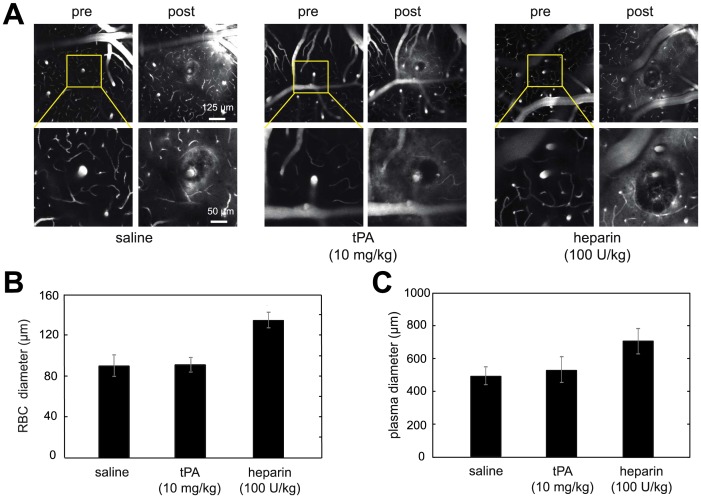
Femtosecond laser-induced cortical microhemorrhage volumes are not increased by i.v. administration of tPA. (**A**) Two-photon excited fluorescence *in vivo* image projections of fluorescently-labeled blood plasma spanning a 20 µm depth centered at the hemorrhage origin. Image stacks are shown before and after rupturing the wall of a single penetrating arteriole using tightly focused femtosecond laser pulses. Representative examples from the three different intravenous infusion groups (saline, tPA, heparin) are shown. Extravasated plasma is visualized by diffuse fluorescence and can be seen in the post hemorrhage images in a halo surrounding the target vessel. The dark core immediately adjacent to the target vessel is filled with red blood cells. Post-hemorrhage (**B**) red blood cell (RBC) and (**C**) blood plasma extravasation diameters (mean±SEM) are shown for the three treatments: saline (n = 9 microhemorrhages), tPA (n = 11), and heparin (n = 10). No difference was found between the saline and tPA group, but heparin significantly increased both red blood cell and blood plasma extravasation diameters.

To rule out the possibility that our findings were caused by artifacts specific to the collagenase model of ICH, we repeated these experiments using an independent ICH model, involving laser-mediated rupture of cerebral blood vessels under in vivo imaging **(see Methods for details).** Cranial windows were implanted in mice (n = 11). Five days later, bleeding was induced in one to four penetrating arterioles per mouse by injuring the targeted endothelium with tightly-focused femtosecond laser pulses [10]. This technique produces hematoma volumes about 20,000 times smaller than the ICH model, but this model provides a powerful way to image, in real time, the development of vascular rupture and bleeding in vivo. During the production of these hemorrhages, animals received intravenous saline (n = 4), tPA (n = 4), or heparin (n = 3), as described above. Using in vivo two-photon excited fluorescence microscopy, we found no difference in the size of the red blood cell (RBC)-filled microhemorrhage core or the surrounding blood plasma-filled region between saline and tPA-treated mice **(** **Fig. 3A–C** **)**. In contrast, hemorrhages produced while infusing heparin were significantly larger than saline controls (ANOVA, post hoc; RBC diameter: p<0.001; plasma extravasation diameter: p = 0.019). We identified 18 of the femtosecond laser-induced intracerebral microhemorrhages in post-mortem tissue sections. Across all treatments, we found that, in addition to making a spherical hematoma, RBCs spread vertically along the perivascular space surrounding the targeted penetrating arteriole **(Fig. S2a–c)**. Blood plasma, however, was able to diffuse through the parenchyma tissue and exhibited a more extended spatial pattern **(Fig. S2d–f)**. We consistently observed that heparin treatment led to a larger spherical RBC-filled volume compared to saline and tPA treatments **(Fig. S2c and S2f)**.

**Figure 4:**
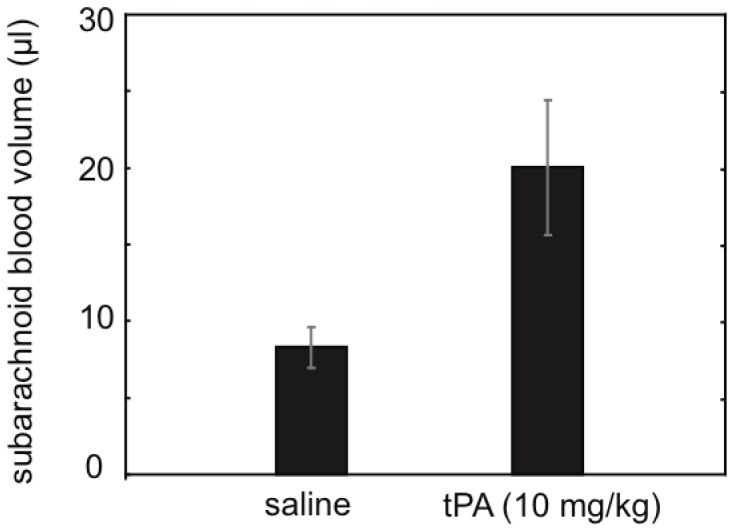
Treatment with tPA worsened subarachnoid hemorrhage. Blood volumes at 24 hrs after SAH induction (mean±SEM) are provided. tPA significantly increased SAH blood volume (tPA, n = 6; saline, n = 7).

Our results showed that tPA did not worsen bleeding in two independent animal models of ICH. In order to further assess the specificity of our findings, we next tested tPA in a pathophysiologically different type of brain injury, subarachnoid hemorrhage (SAH). Following a standard model, SAH was induced in mice by perforating the left anterior cerebral artery using a blunted nylon suture [11]. Thirty min later, saline or tPA was infused, as described above. Subarachnoid blood volume was quantified at 24 hrs using the same photometric assay as before. SAH blood volumes were significantly larger in tPA-treated animals compared to saline controls (mean±SD 20.0±10.8 vs. 8.3±3.5 µl, p = 0.010, t-test) **(** **Fig. 4** **)**. SAH severity was clearly worsened by tPA as 3 out of 6 tPA-treated mice died within 24 hrs, compared to 0 out of 7 saline animals. Functional outcome assessed on an ordinal scale was also significantly worsened in tPA-treated SAH mice (p = 0.014, Mann Whitney U-test).

**Figure 5:**
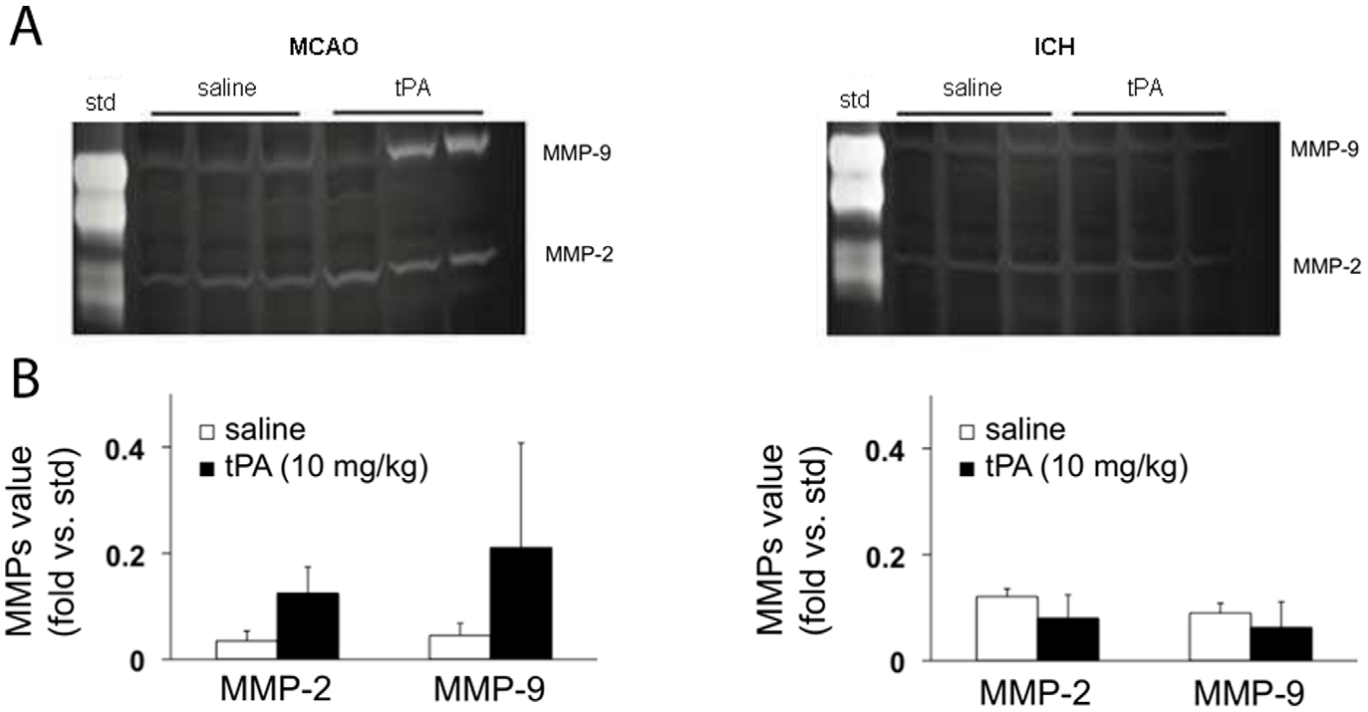
Gelatin zymography of MMP responses. (**A**) Representative zymogram gels showing MMP-2 and MMP-9 levels in brain homogenates derived from mice subjected to focal cerebral ischemia (induced by middle cerebral artery occlusion, MCAO) or primary ICH mice. (**B**) Quantified densitometry of the zymogram results (arbitrary units normalized to constant loading of MMP standards). After 3 hrs of focal cerebral ischemia followed by 3 hrs reperfusion, tPA significantly amplified MMP-2 and MMP-9 levels. In contrast, there were no differences in MMPs in saline versus tPA-treated mice after ICH.

It is known that tPA can exacerbate neurovascular injury during cerebral ischemia. Indeed, many labs have shown that administration of tPA worsens blood brain barrier damage during reperfusion injury in ischemic stroke, in part by amplifying matrix metalloproteinases (MMPs) [12], [13]. Hence, we hypothesized that the different outcomes after tPA application in focal cerebral ischemia versus ICH may be accompanied by different responses in MMP activity. Mice were subjected to either transient focal cerebral ischemia or collagenase-induced ICH and randomized into saline or tPA groups. For the ischemia group, the middle cerebral arteries were occluded with intraluminal sutures for 3 hrs, then mice were treated with saline or 10 mg/kg tPA intravenously, followed by reperfusion via withdrawal of the occluding sutures. Brains were extracted and MMP levels were measured after 3 hrs of reperfusion. For the ICH group, mice were treated with saline or 10 mg/kg tPA intravenously at 3 hrs after collagenase injection, then brains were extracted for MMP measurements at 6 hrs. As expected, MMP-2 and MMP-9 levels were elevated in injured ipsilateral brain tissue in all ischemia and hemorrhage mice. In focal cerebral ischemia, MMP levels were significantly higher in tPA-treated mice compared to saline-treated mice. However, tPA did not amplify MMPs in brains from ICH mice **(** **Fig. 5A–B** **, Fig. S3)**.

## Discussion

Our results in two different mouse models of ICH suggest that intravenous tPA application during the phase of hematoma formation does not increase intracerebral hemorrhage volume. Our findings stand in sharp contrast to the widespread – but so far unproven – assumption that tPA would enlarge hematomas if inadvertently given to patients with acute ICH. Unlike tPA, the anticoagulant heparin significantly increased intracerebral hemorrhage volume. This positive control demonstrates that both of our ICH models are able to detect hematoma enlargement if it occurs. And the results are consistent with our previous findings that another anticoagulant, warfarin, exacerbates hemorrhage in ICH mice as compared to anticoagulation-naïve controls [8]. Although we did not quantify absolute fibrinolytic activity in comparison to reference standards, we showed that our tPA preparations were thrombolytically active, both in-vivo and in-vitro.

From a mechanism standpoint, the massive hematoma enlargement after heparin therapy suggests that drugs were administered prior to definitive clot formation. Unlike warfarin and heparin, tPA has no effect on primary hemostasis and does not delay initial coagulation. But as an activator of the fibrinolytic cascade, tPA increases the risk of re-bleeding from previously hemostatic stable areas [14]. So tPA could theoretically worsen intracerebral bleeding. On the other hand, the fibrinolytic activity of tPA is highly dependent on parameters such as clot structure. For example, platelet-rich thrombi are much less susceptible to tPA dissolution compared with erythrocyte-rich thrombi, mainly due to the release of platelet-derived plasminogen activator inhibitor [15]. Intracerebral hematomas consist of whole blood containing large amounts of platelets so it is possible that these structures may be more tPA-resistant. Another possible explanation for our finding is that the ICH clots surrounding ruptured brain blood vessels are heterogeneous and larger than singular intra-arterial thrombi in ischemic stroke [9]. Standard doses of tPA may not be able to dissolve significant parts of the coagulated hematoma in order to induce re-bleeding. Furthermore, ICH may be stabilized by the counterpressure of surrounding brain tissue, which limits hematoma expansion. Elevated pressure in the brain areas near the hematoma may even prevent systemic intravenously administered tPA from reaching the initial sites of vessel rupture.

Our finding that tPA does not amplify MMP-2 and MMP-9 in ICH potentially suggests that this drug is not activated by a parenchymal (i.e. extra-arterial) clot formation in the same way as it is activated by an intraluminal thrombus in ischemic stroke [16]. However, there might be other members of the MMP family involved in the pathophysiologic cascades following ICH that are activated by tPA treatment [17]. Brain edema was not measured in our study. But tPA was found to aggravate brain edema if injected into the ventricles in the case of ICH with ventricular extension (in this setting, tPA injection is intended to restore the drainage of the cerebrospinal fluid) [18], [19]. Nevertheless, it is unlikely that differences in edema formation between tPA- and saline-treated mice have influenced our main outcome parameter (i.e., ICH volume), as hematoma expansion and edema formation occur sequentially rather than simultaneously.

In contrast to ICH, our results clearly show that tPA dramatically increases blood volume in SAH. Pathophysiologically, SAH results from the rupture of a larger intracranial artery in the subarachnoid space outside the brain. Blood extravasation is immediately followed by the induction of the coagulation cascade, leading to the formation of a fibrin-rich clot in the vessel wall [20]. In ischemic stroke, tPA is able to dissolve thrombi in arteries of similar diameter [1]. Thus, it is conceivable that tPA applied in acute SAH is able to reach the site of the initial vessel rupture, thereby dissolving newly-formed arterial clots. Any re-bleeding would then easily expand into the subarachnoid space.

What is extremely important is that our results must be distinguished from the fact that tPA therapy increases the risk of hemorrhagic transformation after ischemic stroke [21]. The mechanisms involved in primary ICH are different from those that underlie hemorrhagic transformation and reperfusion injury after ischemia. Hemorrhagic transformation during reperfusion injury is now known to be related to excitotoxic effects of tPA and neurovascular upregulation of MMPs [5], [12], [13], rather than the re-bleeding of a previously terminated hematoma. tPA may not worsen ICH, but this is different from potential deleterious effects of tPA during cerebral ischemia and reperfusion. Our present data support this idea. tPA seems to amplify MMPs during cerebral ischemia-reperfusion but not during ICH formation.

We performed an extensive literature research, but did not find clinical or experimental data targeted to explore the effects of tPA on hematoma volume in the acute phase of ICH. However, a recent study on memantine as an adjunctive therapy to tPA in ischemic stroke also analyzed a group of mice subjected to collagenase ICH. [22] Whereas a bolus injection of tPA (10 mg/kg) 30 min after ICH induction increased hematoma size on MRI scans, the combined administration of tPA and memantine did not change ICH as compared to controls. As memantine is a NMDA-antagonist which does not reduce the thrombolytic activity of tPA itself, these findings are worthwhile to be further explored in the context of the present study. In addition, we found one more study that explored tPA administration in a guinea pig model of collagenase ICH. [23] In this work, a dose-dependent increase of hematoma volumes was observed. The results of the higher dose groups stand in apparent contrast to our findings and need further evaluation.

One should be cautious when transferring our experimental findings to the clinical setting. Despite testing our hypothesis in two pathophysiologically independent models of ICH, significant species differences and model limitations may play a role. In particular, the collagenase model represents “deep” hemorrhage formation occurring in or nearby the basal ganglia, while the laser-rupture model more closely represents cortical microhemorrhages, with a diameter of only 100 µm. It is difficult to extrapolate from these models to, for example, large cortical hemorrhages in humans. The latter frequently result from the rupture of pathological vessels, for example arteriovenous malformations or vessels with cerebral amyloid angiopathy, and our models of bleeding from normal vessels cannot rule out differences in the effects of tPA due to pre-existing vessel pathology. Furthermore, the coagulation system of mice and humans are not entirely comparable, as some murine factors have different roles than their human counterparts [24]. Of course, if it would be true that intravenous tPA does not enlarge hematomas or worsen outcomes in ICH, then tPA could be administered at much earlier time points to patients exhibiting signs of stroke. This could vastly increase the effectiveness of this drug in patients with ischemic stroke, while not worsening outcome for the patients with ICH. However, based on clinical findings alone, it is not always possible to distinguish patients with SAH from those with ICH. Some SAH may mimic ICH, due to a parenchymal extension of the primary bleeding into the lobes that surround the aneurysm [25]. According to our findings, treating a SAH patient with tPA would have negative sequelae.

In summary, our experimental data do not support the assumption that intravenous tPA application significantly increases hematoma volume in acute ICH. Due to species differences and technical limitations of the models used, our findings cannot be immediately transferred to a clinical setting. More clinical and experimental studies are warranted.

## Methods

All experiments were performed under protocols approved by the Massachusetts General Hospital Institutional Animal Care and Use Committees (IACUC, protocol N-00087) and the Cornell Institutional Animal Care and Use Committees (IACUC, protocol 2009–0043) following the NIH Guide for Use and Care of Laboratory Animals. The ARRIVE guidelines were followed (please see checklist below).

### In-vitro tPA Activity Assay and In-vivo Reperfusion Studies

A 96-well plate was pre-loaded with mouse plasminogen (10 µg/ml) and the fluorogenic plasmin substrate D-Val-Leu-Lys-7-Amino-4-Methylcoumarin (200 µM) in a final volume of 50 µl phosphate buffered saline (PBS). Thereafter, 0.125 µl of human recombinant tPA stock-solution (4 mg/ml) or saline was added. In this assay, tPA converts plasminogen to plasmin, then plasmin converts D-Val-Leu-Lys-7-Amino-4-Methylcoumarin to a fluorescent product, which was measured over 1 hr at 5 min intervals using a pre-warmed fluorescence plate reader (37°), with excitation set at 360 nm and emission at 460 nm. Three measures were performed per group and time point [6].

In-vivo thrombolytic activity of tPA was demonstrated by a standard model of ischemic stroke based on a thromboembolic occlusion of the middle cerebral artery in rats (see [7] for details). In brief, one hour after initiation of ischemia, 4 animals per group were treated intravenously with either saline or tPA (10 mg/kg). Laser-Doppler flowmetry was used to monitor regional cerebral blood flow for up to 150 min after treatment.

### Mouse Model of Collagenase-induced Intracerebral Hemorrhage

#### Experimental procedures

Experiments were performed in male CD-1 mice (12–16 weeks) under isoflurane anesthesia, following an institutionally approved protocol (Massachusetts General Hospital N-00087) and in accordance with the National Institute of Health’s Guide for the Care and Use of Laboratory Animals. Under isoflurane anesthesia (1.5% to 2%) with spontaneous respiration in a nitrous oxide–oxygen mixture, the right jugular vein was prepared. After ligating the distal part of the vessel, a PE-10 tube was inserted into the jugular vein and moved forward in the proximal direction. After properly fixing the tube and closing the suture, the animals were placed in a stereotactic frame. Under ongoing anesthesia, a small borehole was drilled, and a 32-gauge 0.5 µl microinjection needle (Hamilton, 7000 series, Hamilton, Reno, NV, USA) was slowly lowered into the right striatum at the following stereotactic coordinates from bregma: 0.0 mm anterior, 2.0 mm lateral, and 3.5 mm depth. Over a period of 5 min, 0.5 µl of saline containing 0.2 U of collagenase VII-S (Sigma-Aldrich, St Louis, MO, USA) was injected [8], [9]. The needle was left in place for 10 min, and then slowly removed over a period of 5 min. Thereafter, the borehole was sealed with bone wax and the scalp closed. The mice were then allowed to wake up and were put in a mouse restrainer under light restraint. Beginning 30 min or 4 hrs after hemorrhage induction, respectively, saline (500 µl), recombinant human tPA (10 mg/kg, diluted in 500 µl saline) or heparin (100 U/kg, diluted in 500 µl saline) were continuously injected through the jugular vein catheter for 30 min using an injection pump (10% bolus injection over the first minute). The correct intravenous position of the catheter was double-checked by drawing some blood into the tube after the injection. The catheter was then ligated and cut near the skin and the mice were returned to their cages.

#### Functional outcome and ICH volume determination

Twenty-four hrs after hemorrhage induction, mortality rate was determined and functional outcome was assessed using a standard hanging wire test [8]. Afterwards, mice were perfused with PBS and sacrificed under deep anesthesia. Brains were removed, separated into left and right hemispheres, and placed in glass tubes containing 3 ml PBS. After 30 sec of homogenization, ultrasound was applied for 1 min. After centrifugation for 30 min (13 000 rpm, 4°C), 250 µl supernatant was added to 1 ml Drabkins reagent. With use of a photometer, absorption rates were determined at 540 nm, and hematoma volumes were calculated for the entire brain (both hemispheres) on the basis of a standard curve [8]. Mice that died within the first 24 hrs after ICH induction could not undergo perfusion. In these cases, we subtracted 2.1 µl from the total blood volume derived from the hemoglobin assay. This volume was found to be the mean difference in blood content between perfused and non-perfused brains [8].

#### Determining the kinetics of hematoma development

In order to assure that the medication is administered during hematoma formation, we sacrificed untreated mice under deep anesthesia at 30 min (n = 3), 60 min (n = 4) and 24 hrs (n = 1) after hemorrhage induction. Brains were removed and cut in slices using a 1-mm matrix to visually identify the location and size of the hematoma **(** **Fig. 2A** **)**. All mice showed bleeding in the right striatum. Together with previous studies using the collagenase model [8], these data suggested that the time interval of treatment administration (i.e., 30 to 60 min after hemorrhage induction) well matches the kinetics of hematoma formation.

### Mouse Model of Laser-induced Parenchymal Microhemorrhage

#### Experimental procedures

All animal procedures were approved by Cornell University Institutional Animal Care and Use Committee, following an institutionally approved protocol (2009–43) in accordance with the National Institute of Health’s Guide for the Care and Use of Laboratory Animals. Eleven C57BL/6J mice (The Jackson Laboratory; 5 female, 6 male) ranging from 21 to 32 g in mass were used to image the size of femtosecond laser induced hemorrhages. To prepare a chronic cranial window for imaging, mice were anesthetized using 5% isoflurane (VetOne) and maintained at 1.5–2%. Body temperature was kept at a constant temperature of 37.5°C with a heating blanket and thermometer (50-7053P; Harvard Apparatus). Glycopyrrolate (0.005 mg/kg mouse (Baxter Healthcare Corp.)) was administered intramuscularly while ketoprofen (5 mg/kg mouse (Fort Dodge, Inc.)) and dexamethasone sodium phosphate (0.2 mg/kg mouse (American Regent, Inc.)) were both administered subcutaneously prior to surgery. A 6 mm diameter bilateral craniotomy was performed over the parietal cortex. An 8 mm glass cover slip (Deckglaser; WPI) was then glued over the exposed brain using cyanoacrylate and dental cement (Lang Dental Mfg Co.). Animals were administered 5% (wt/vol) glucose in physiological saline (0.1 ml/100 g mice) subcutaneously and gradually transitioned off isoflurane anesthesia. Mice were then administered ketoprofen (5 mg/kg) every 24 hrs for 72 hrs and allowed a minimum of five days recovery.

#### Two-photon excited fluorescence microscopy

Before imaging sessions, mice were anesthetized with isoflurane and the jugular vein was cannulated, as described above. Mice were intravenously injected with 0.1 ml of 5% (wt/vol) lysine-fixable Texas-red dextran (70 kDa) fluorescent dye (D1864; Invitrogen) in physiological saline to label the vasculature. In-vivo imaging was conducted with a locally-designed two-photon excited fluorescence (2PEF) microscope using a 1045-nm, 1-MHz, 350-fs pulse train from a Yb-fiber oscillator/amplifier system (µJewel FCPA, IMRA America, Inc.) as the excitation source. Fluorescence emission was collected with a 65 nm wide filter centered at 645 nm (Chroma). 2PEF imaging revealed surface blood vessels and sub-surface capillaries (Fig. 3A). In addition to visualizing vascular topology, we measured the diameter and blood flow speed and direction in individual vessels, as described previously [26]. For hemorrhages, we targeted cortical penetrating arterioles (PA), i.e. arterioles that branch from the surface arteriole network and dive into the brain to feed capillary beds [27]. These vessels were identified by tracing through the vascular network from readily-identifiable surface arterioles and we confirmed they were penetrating arterioles by checking that the blood flow direction was into the brain.

#### Penetrating arteriole microhemorrhage by femtosecond laser ablation

Each mouse was subjected to one to four microhemorrhages with a minimum of 1 mm spacing between hemorrhaged vessels. 2PEF image stacks of each microhemorrhage site (**Fig. 3A**) as well as diameter and blood flow speed measurements of the target PAs were collected before microhemorrhages were induced. A 50 µl bolus injection of saline (9 microhemorrhages across 4 animals), tPA (11 microhemorrhages across 4 animals), or heparin (10 microhemorrhages across 3 animals) was infused through the jugular vein catheter, then after five minutes a constant infusion (18 µl/min for 25 min) was set using a syringe pump (PHD2000; Harvard Apparatus), as described above.

During the period of steady infusion, the targeted PAs were once again located and microhemorrhages were produced in the descending segment of the PA, about 50–100 µm below the surface of the brain. To induce a microhemorrhage, 50 fs duration laser pulses were tightly focused (1.0 numerical aperture) on the wall of the targeted PA, as described by Nishimura, et al [10]. Briefly, a laser energy of about 700 nJ was initially applied for a 10 pulse burst (1-kHz repetition rate). If the vessel did not rupture, the laser energy was increased by about 50% and the vessel was irradiated again. This process was repeated until extravasation of red blood cells (RBC) and blood plasma into the parenchyma of the brain was observed. The variation of required laser energy to trigger a microhemorrhage depends mainly on the depth of the target vessel beneath the brain surface and the presence or absence of large blood vessels on the brain surface above the target vessel. We use the minimum laser energy required to rupture the vessel wall of the targeted PA and initiate bleeding into the brain. 2PEF image stacks were taken immediately and 30 min after each PA hemorrhage (**Fig. 3A**).

#### Red blood cell and plasma diameter measurements

During the microhemorrhage, RBCs and plasma exit the lumen of the targeted PA. Since the fluorescent marker only labels blood plasma, under 2PEF imaging the RBCs are visualized as dark patches in a sea of fluorescently-labeled blood plasma leaving the vessel. After the hemorrhage, the region immediately surrounding the targeted PA is filled with RBCs [10]. This region appears dark in the 2PEF image because the RBCs are densely packed and largely exclude the fluorescently-labeled blood plasma (**Fig. 3A**). Blood plasma exiting the lumen of the target vessel moves further into the brain parenchyma than the RBCs and can be visualized by a ring of increased fluorescence surrounding the RBC filled hemorrhage core. RBC hemorrhage and plasma extravasation diameters were calculated from a maximal projection of 20 µm of the 2PEF image stack (ImageJ) taken 30 min after the microhemorrhage, with the stack centered on the depth where the microhemorrhage was created. The researcher making the diameter measurements was blinded to the treatment group.

#### Post-mortem histology

At the end of each imaging session, animals were transcardially perfused with 30 ml of phosphate buffered saline (PBS) (Sigma-Aldrich) and 100 ml of 4% (wt/vol) paraformaldehyde (Fisher Scientific) in PBS. The brain was extracted from the skull and cryoprotected by immersion in 30% (wt/vol) sucrose in PBS for 24 hrs and then in 60% sucrose in PBS. Fiducial marks were made in the left corners of the craniotomy window by vertically inserting a 25-gauge needle 2 mm into the brain. The needle was externally tinted with black ink (Parker). The fiducial marks were placed in known locations relative to the microhemorrhage sites. The brain was then frozen and cut into 50 µm thick coronal sections on a cryostat. The sections were mounted onto microscope slides (Superfrost Plus, Fisher Sci.) and incubated for 12 min with diaminobenzadine (DAB) (Peroxidase Substrate kit, Vector labs, No. SK-4100) to stain endogenous peroxidase in RBCs and then rinsed with deionized water. The sections were photographed under brightfield and fluorescence microscopy. Fiducials were mapped and used to identify the slices and location within slices where microhemorrhages were located. Microhemorrhages were identified on the basis of both DAB stained RBCs and Texas red-dextran fluorescence in the parenchymal space **(Fig. S2A–C)**.

#### Penetrating arteriole volume blood flux and laser energy used to make hemorrhage do not influence hemorrhage sizes

We tested whether there was any correlation between the measured RBC and plasma extravasation diameters and the volume blood flux of the targeted PA or the laser energy used to trigger the microhemorrhage. Within the range of laser energies and PA blood fluxes studied here, there were no significant correlations between the RBC and plasma diameters with penetrating arteriole flux rates or deposited energy across all three treatment groups (Pearson product-moment correlation coefficient for RBC hemorrhage diameter, Spearman’s rank correlation coefficient for plasma diameter).

### Mouse Model of Subarachnoid Hemorrhage

#### Experimental procedures

In male CD-1 mice, isoflurane anesthesia (1.5% to 2%) was induced with spontaneous respiration in a nitrous oxide–oxygen mixture. A heating pad was used to maintain the body temperature at 37.0±0.5°C. A blunted 4–0 monofilament nylon suture was introduced into the external carotid artery and advanced through the internal carotid artery to the left anterior cerebral artery (ACA) near the anterior communicating artery, where resistance was encountered and a severe decrease was seen in cerebral blood flow. The filament was advanced 3 to 5 mm further to perforate the ACA, then immediately withdrawn. SAH was confirmed by the persistence of reduction in CBF [11]. Then, the right jugular vein was prepared and the catheter for drug infusion was inserted as described above. Thirty min after SAH induction, saline or tPA was infused as described above.

#### Functional outcome and quantification of subarachnoid blood volume

Twenty-four hrs after SAH induction, mortality and functional outcome was assessed using a modified 18-point scoring system in a blinded fashion [28]. Subarachnoid blood volume was also assessed by means of a previously published photometric hemoglobin assay method, with slight modifications [28], [29]. In brief, mice were perfused using PBS and sacrificed under deep anesthesia. Brains were dissected and removed with the dura intact to avoid the loss of any subarachnoid blood, and whole brain placed in glass tubes containing 3 ml PBS. Hemoglobin content was measured as described above. Mice that died within the first 24 hrs after SAH induction could not undergo perfusion. In these cases, we subtracted 2.1 µl from the total blood volume derived from the hemoglobin assay, as described above.

### SDS-PAGE Gelatin Zymography

Gelatin zymograms were used to measure the levels of MMP-2 and MMP-9 in brain homogenates from ICH mice and from mice that underwent focal cerebral ischemia as described elsewhere in detail [30], [31]. For the ICH group, mice were treated with saline (n = 5) or 10 mg/kg tPA (n = 5) intravenously at 3 hrs after collagenase injection. Following transcardial perfusion with ice-cold PBS under deep anesthesia, brains were extracted at 6 hrs after ICH induction. For the ischemia group, the middle cerebral arteries were occluded with intraluminal sutures for 3 hrs. At the time point of reperfusion, mice were treated with saline (n = 5) or 10 mg/kg tPA (n = 5) intravenously. Brains were extracted after 3 hrs of reperfusion. Brains were divided into ipsilateral and contralateral hemispheres, then frozen immediately in liquid nitrogen and stored at −80°C. Samples were homogenized in lysis buffer including protease inhibitors on ice. After centrifugation, supernatant was collected, and total protein concentrations were determined using the Bradford assay (Bio-Rad). Prepared protein samples were loaded and separated by 10% Tris-glycine gel with 0.1% gelatin as substrate. MMP activity was quantified via standard densitometry. MMP indexes were calculated based on purified MMP-2 and -9 standards.

### Statistical Analysis

Statistical analysis was performed using SPSS 19.0 (IBM, Armonk, NY) and StatsDirect v.2.7.8 (Cheshire, UK). Data were tested for normality and variance homogeneity. We used one-way ANOVA to determine if there were differences across the three treatment groups. If statistically significant differences were found by ANOVA, we performed pair-wise comparisons between groups using the Bonferroni multiple-comparisons correction. Depending on scale level and data distribution, t-test or Mann Whitney U-test were used to compare means between two groups, respectively. p-values less than 0.05 were considered statistically significant.

## Supporting Information

Figure S1 Functional outcome 24 hrs after ICH induction was assessed by means of a standard hanging wire test (time to fall-off, maximum 60 sec, three attempts per mouse). No difference was observed between saline- and tPA-treated mice.
(TIF)

Figure S2 Representative coronal post-mortem tissue sections of microhemorrhages from animals treated with saline **(a, d)**, tPA **(b, e)** or heparin **(c, f)**. Panels **(a-c)** show white-light transmission images of DAB treated sections, which show the RBCs that have hemorrhaged into the tissue as black. Panels **(d-f)** show fluorescence images of extravasated Texas Red-dextran for the same sections as panels (a-c).
(TIF)

Figure S3 Gelatin zymography of MMP responses. Representative zymogram gels showing MMP-2 and MMP-9 levels in brain homogenates derived from middle cerebral artery occlusion (MCAO) **(A)** or primary ICH **(B)** mice. In both MCAO and ICH models, MMP-2/−9 levels on the contralateral side were not affected by tPA treatment, indicating that tPA treatment does not change MMP-2/−9 baseline levels. In contrast, in MCAO mice, MMP levels on the ipsilateral side were increased by tPA treatment (see **Fig. 5** for more details).
(TIF)
