# Supplementary figures and images for: Intravenous tPA Therapy Does Not Worsen Acute Intracerebral Hemorrhage in Mice
Source: PLoS One. 2013 Feb 8;8(2):e54203. doi: 10.1371/journal.pone.0054203 (PMC3568130; doi:10.1371/journal.pone.0054203)

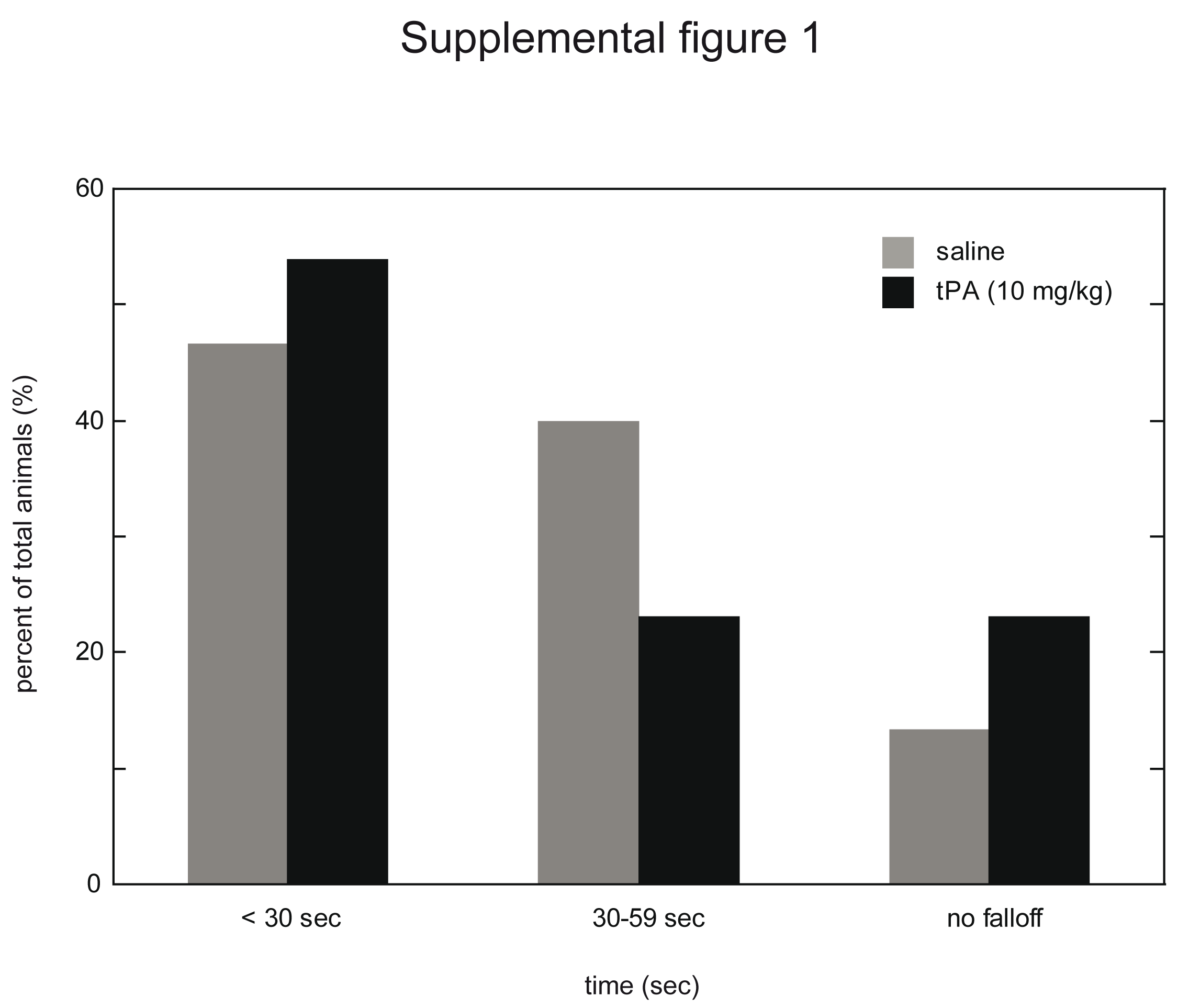

Supplement: Figure S1 — Functional outcome 24 hrs after ICH induction was assessed by means of a standard hanging wire test (time to fall-off, maximum 60 sec, three attempts per mouse). No difference was observed between saline- and tPA-treated mice. (TIF) [file pone.0054203.s001.tif]

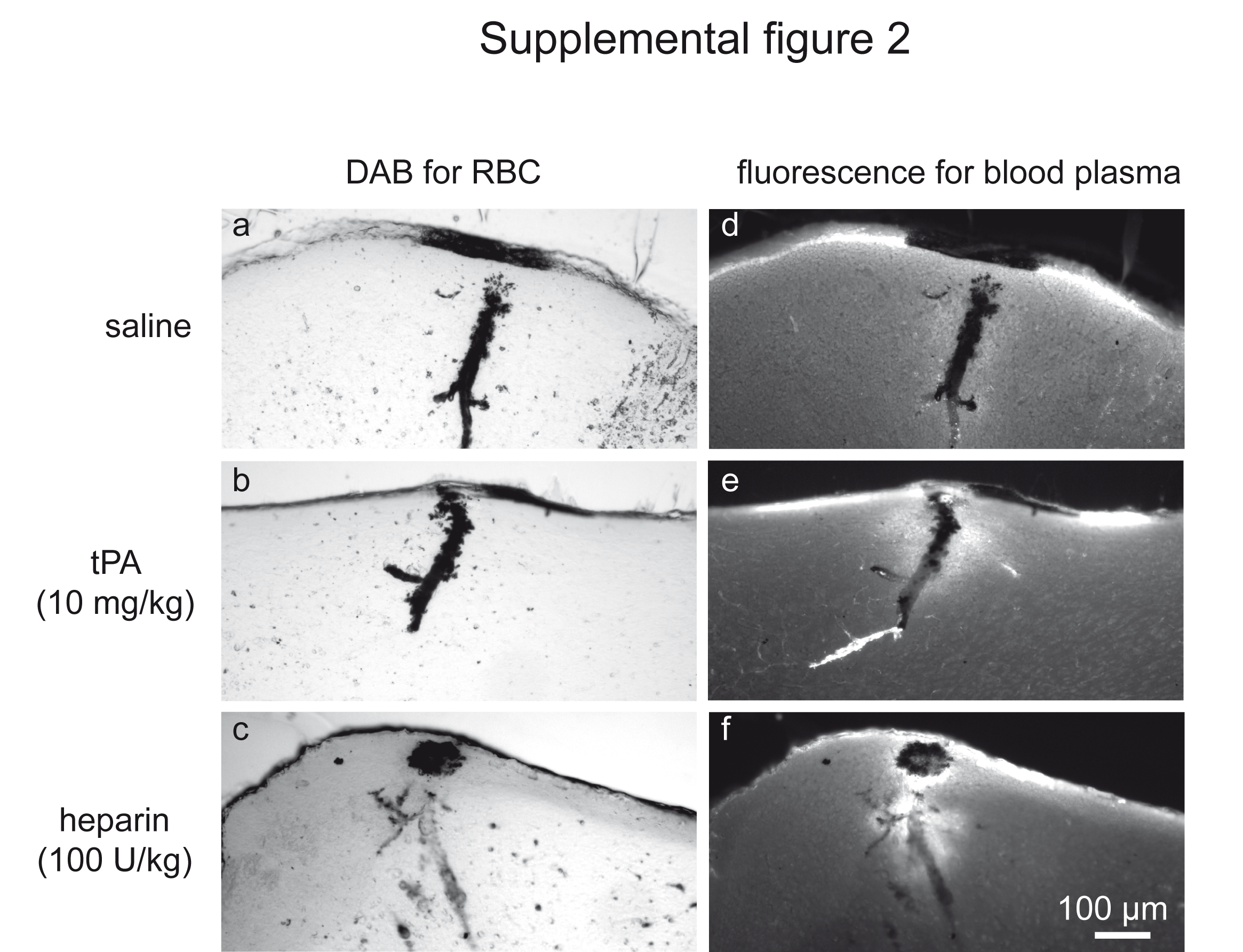

Supplement: Figure S2 — Representative coronal post-mortem tissue sections of microhemorrhages from animals treated with saline (a, d), tPA (b, e) or heparin (c, f). Panels (a-c) show white-light transmission images of DAB treated sections, which show the RBCs that have hemorrhaged into the tissue as black. Panels (d-f) show fluorescence images of extravasated Texas Red-dextran for the same sections as panels (a-c). (TIF) [file pone.0054203.s002.tif]

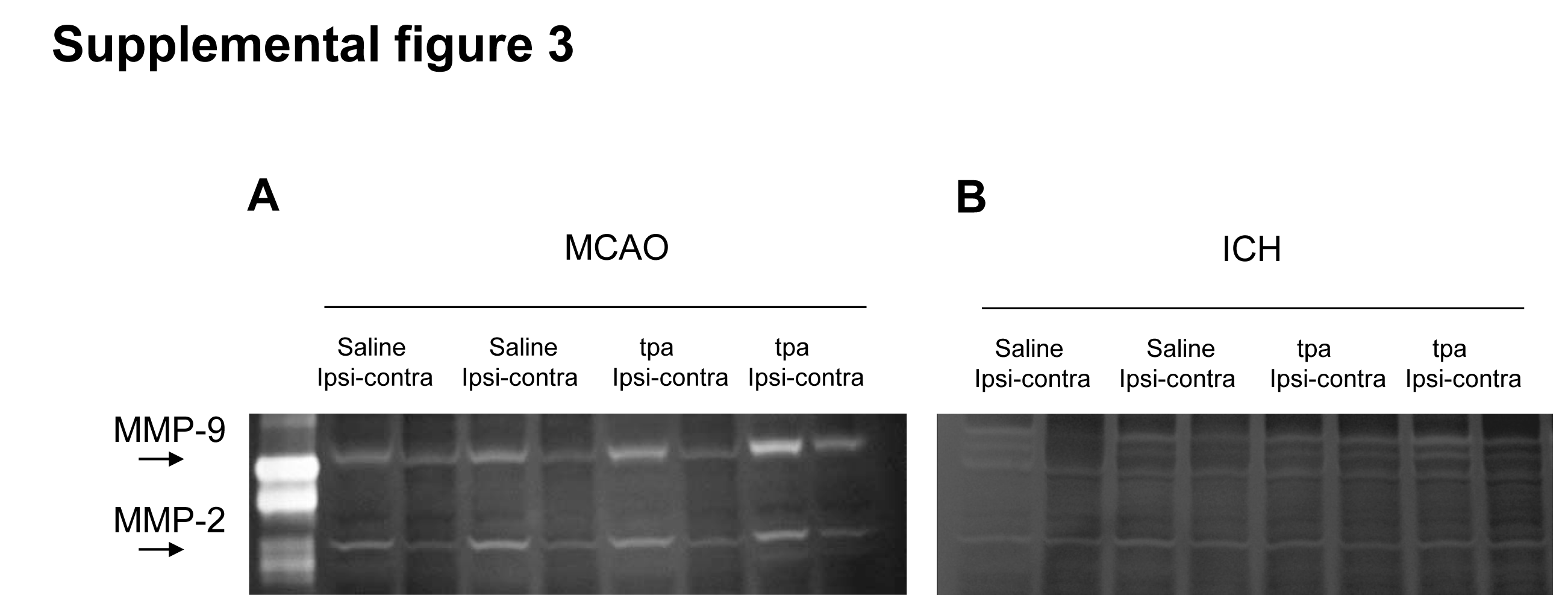

Supplement: Figure S3 — Gelatin zymography of MMP responses. Representative zymogram gels showing MMP-2 and MMP-9 levels in brain homogenates derived from middle cerebral artery occlusion (MCAO) (A) or primary ICH (B) mice. In both MCAO and ICH models, MMP-2/−9 levels on the contralateral side were not affected by tPA treatment, indicating that tPA treatment does not change MMP-2/−9 baseline levels. In contrast, in MCAO mice, MMP levels on the ipsilateral side were increased by tPA treatment (see Fig. 5 for more details). (TIF) [file pone.0054203.s003.tif]
